# Familial hypercholesterolemia class II low-density lipoprotein receptor response to statin treatment

**DOI:** 10.1242/dmm.042911

**Published:** 2020-04-06

**Authors:** Linda Omer, Lubna Hindi, Giuseppe Militello, Katlin B. Stivers, Kenneth C. Tien, Nolan L. Boyd

**Affiliations:** 1 Cardiovascular Innovation Institute, University of Louisville, Louisville, KY 40202, USA; 2 Department of Biochemistry and Molecular Genetics, University of Louisville, Louisville, KY 40202, USA; 3 Department of Bioengineering, University of Louisville, Louisville, KY 40202, USA; 4 Department of Microbiology and Immunology, University of Louisville, Louisville, KY 40202, USA; 5 Department of Physiology, University of Louisville, Louisville, KY 40202, USA

**Keywords:** Familial hypercholesterolemia, Low-density lipoprotein, Cholesterol, Endoplasmic reticulum stress, Clustered regularly interspersed palindromic repeats

## Abstract

Low-density lipoprotein (LDL) receptor (LDLR) mutations are the primary cause of familial hypercholesterolemia (FH). Class II LDLR mutations result in a misfolded LDLR retained in the endoplasmic reticulum (ER). We have developed a model of FH class II and CRISPR-corrected induced pluripotent stem cells (iPSC) capable of replicating mutant and repaired LDLR functions. We show here that iPSC and derived hepatocyte-like cells (HLC) replicate misfolded LDLR accumulation and restoration of LDLR function in CRISPR-corrected cells. It was reported that model cells overexpressing class II LDLR mutants result in endoplasmic reticulum (ER) accumulation of immature LDLR and activation of the unfolded protein response (UPR). We show here that statins induce a similar accumulation of immature LDLR that is resolved with class II correction. We also demonstrate that, although capable of UPR induction with tunicamycin treatment, unlike overexpression models, statin-treated class II iPSC and derived HLC do not induce the common UPR markers Grp78 (also known as HSPA5) or spliced XBP1 [XBP1 (S)]. Because statins are reported to inhibit UPR, we utilized lipoprotein-deficient serum (LPDS) medium, but still did not detect UPR induction at the Grp78 and XBP1 (S) levels. Our study demonstrates the recapitulation of mutant and corrected class II LDLR function and suggests that overexpression models may not accurately predict statin-mediated class II protein biology.

## Abstract (precis)

**Summary:** Statin-induced upregulation of class II mutant LDLR does not cause acute endoplasmic reticulum (ER) stress and unfolded protein response induction, in spite of misfolded LDLR protein accumulation in the ER.

## INTRODUCTION

Familial hypercholesterolemia (FH) is an autosomal dominant disease primarily caused by mutations in the low-density lipoprotein (LDL) receptor (*LDLR*) gene, leading to premature cardiovascular disease (CVD) (Gidding, 2016; Goldstein and Brown, 2009; Varret and Rabes, 2012). The LDLR activity level affects disease severity and is based on which mutations are present (Hobbs et al., 1990). There are over 1200 LDLR mutations identified that are categorized into five classes (Varret and Rabes, 2012). As of 2012, greater than 50% have been described as class II or transport-defective mutations (Gent and Braakman, 2004; Hobbs et al., 1990). After translation, a newly synthesized and unfolded LDLR is processed in the endoplasmic reticulum (ER) as a partially glycosylated precursor of 120 kDa (Gent and Braakman, 2004), aided by the ER chaperones glucose-regulated protein 78 (Grp78; also known as HSPA5), receptor-associated protein (RAP; also known as LRPAP1) and mesoderm development (MESD) (Bu and Schwartz, 1998; Culi and Mann, 2003; Gent and Braakman, 2004; Li et al., 2002). The LDLR is then transported to the Golgi, where N- and O-linked sugars are added, increasing the molecular mass to 160 kDa (Esser and Russell, 1988; Tolleshaug et al., 1983). Class II mutations are commonly referred to as a ‘folding or conformational disease’ (Gent and Braakman, 2004), because these mutations result in a misfolded LDLR that is either unable or has a less than 5% rate of leaving the ER for the Golgi (Hobbs et al., 1990).

The molecular mechanisms responsible for LDLR folding and maturation are still unclear. The quality control system of the ER ensures that newly synthesized proteins only leave the compartment when their folding criteria have been met (Kaufman, 2004). Chaperones expressed in the ER play a significant role in the protein folding process (Ellgaard and Helenius, 2003; Zhang and Wang, 2016). General chaperone Grp78 transiently binds the LDLR and aids in its proper folding under normal conditions (Dorner et al., 1993; Jørgensen et al., 2000; Sørensen et al., 2006). This correlates with studies of Grp78 acting selectively in retaining proteins in the ER (Dorner et al., 1993). This quality control ensures that only properly folded proteins exit the ER for the Golgi, whereas misfolded proteins are retained in the ER for further processing. If these proteins are unable to be corrected, misfolded proteins in the ER can accumulate and cause ER stress, activating the unfolded protein response (UPR) (Gardner et al., 2013; Hetz et al., 2011; Schröder and Kaufman, 2005). The major role of the UPR is to maintain protein homeostasis in the presence of accumulated un/misfolded proteins. The three major stress sensor pathways in UPR activation are inositol-requiring transmembrane kinase/endonuclease (IRE1α), PKR-like ER kinase (PERK; also known as EIF2AK3) and activating transcription factor 6 (ATF6) (Hetz et al., 2011). The UPR works to alleviate ER stress by upregulating the folding capacity through controlling expression of transcription factors and other downstream targets that specifically mediate protein folding, ER-Golgi trafficking, organelle biogenesis and ER-associated degradation (ERAD) (Hetz et al., 2011).

FH is also classified as a misfolded protein disease (Gent and Braakman, 2004), but there is very limited research into class II LDLR misfolding in FH, primarily because of the accelerated devastating effect the disease has on the cardiovascular system. Evidence presented in the literature suggests that ER stress occurs in FH class II mutations because of accumulating misfolded LDLR (Jørgensen et al., 2000; Sørensen et al., 2006). We previously reported the generation of class II FH induced pluripotent stem cells (iPSC) and permanent correction of the homozygous 3-bp deletion using clustered regularly interspaced short palindromic repeats (CRISPR)/CRISPR-associated 9 (Cas9) (Omer et al., 2017). Here, we utilized this model to investigate the differential response between class II mutant and corrected LDLR in iPSC and derived hepatocyte-like cells (HLC). We further characterize LDLR function in HLC and the effects of misfolded class II LDLR accumulation in iPSC and HLC. Of particular note, in contrast to reports using overexpression models of class II LDLR mutants, statin-mediated accumulation of misfolded protein does not appear to be sufficient to cause ER stress or induction of the UPR. Together, these data demonstrate the ability of iPSC/HLC derived from FH patients to model LDLR function. They also indicate that the downstream effects of statin treatment on class II FH patients may differ from responses induced in overexpression models.

## RESULTS

### LDL cholesterol (LDL-C) internalization is restored in corrected HLC

**Fig. 1.**
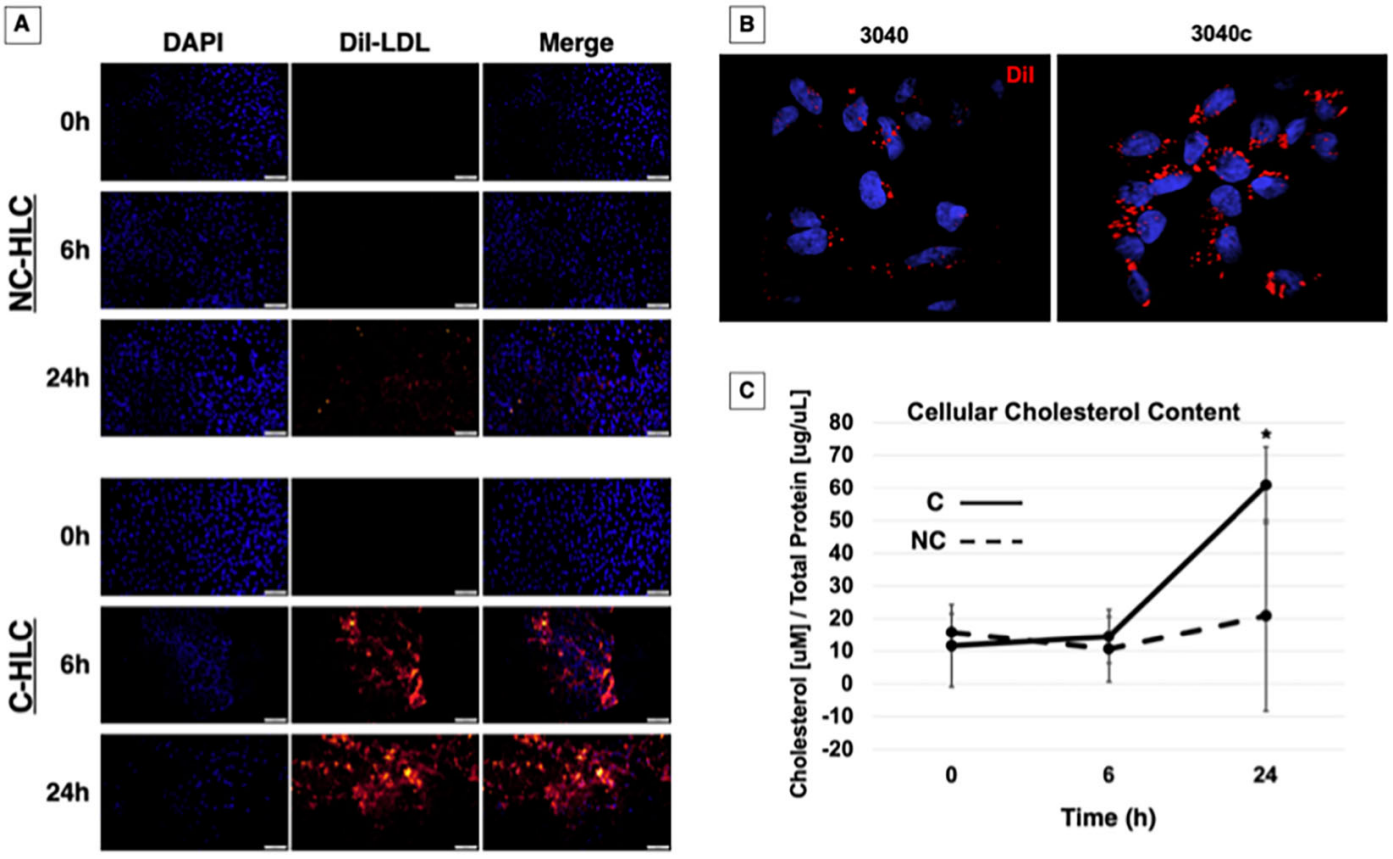
**LDLR-mediated cholesterol internalization is restored in C-HLC.** (A) NC-HLC and C-HLC were treated overnight in 5% LPDS medium supplemented with rosuvastatin. This was followed by incubation with DiI-LDL for 6 h or 24 h. NC-HLC did not show any DiI fluorescence from LDL uptake until 24 h. C-HLC internalized DiI-LDL by 6 h, which increased after 24 h. Scale bars: 100 μm (each image has been linearly brightened to the same level). (B) HLC were imaged at 100× magnification for detection of DiI fluorescence localization. (C) HLC were treated with LPDS medium and rosuvastatin for 48 h followed by incubation in methyl-β-cyclodextrin (MBC) for 45 min when time point 0 h samples were collected. Cells were further incubated for 6 h or 24 h with unlabeled LDL then analyzed for cellular cholesterol concentration with respect to protein. NC-HLC showed no statistical difference in cellular cholesterol for all three time points, demonstrating the dysregulation of cholesterol metabolism in FH HLC. Corrected cells did not have statistically different cholesterol content after 6 h, demonstrating the effect of MBC treatment and plasma membrane cholesterol chelation on receptor-medicated endocytosis. After 24 h exposure to LDL-C, C-HLC were able to increase cellular cholesterol to a statistically significant level [61 µM/(µg/µl) compared to 11.6 µM/(µg/µl) at 0 h]. There was no difference in cholesterol concentration at 0 h between NC-HLC and C-HLC. The graph values represent the mean±s.d. (*n*=3) per treatment, per cell type and replicated three times in the laboratory. Statistics were performed using a two-way ANOVA Holm–Sidak post-hoc test. *P<0.05 compared to NC-HLC at 6 h and 24 h, and C-HLC at 6 h.

Our previously published work was focused on iPSC and H1 human embryonic stem cells (ESC) (collectively referred to as PSC) and the restoration of LDL-C internalization in FH iPSC using CRISPR/Cas9 (Omer et al., 2017), which is potentially important for studying the effects of statins during early development and fetal malformation (Avis et al., 2009; Kusters et al., 2012). Since the liver hepatocyte is responsible for cholesterol regulation, we wanted to examine a time course for replenishment of LDL-C and whether or not the non-corrected (NC)-HLC could recover over a 24-h period by non-receptor mediated mechanisms. We treated HLC overnight in lipoprotein-deficient serum (LPDS) medium with rosuvastatin (0 h), then added 10 µg/ml 1,1′-dioctadecyl-3,3,3′,3′-tetramethyl-indocarbocyanine perchlorate (DiI) fluorescently labeled LDL (DiI-LDL) for 6 h or 24 h. As expected, at 0 h, no DiI fluorescence was detected in either NC-HLC or corrected (C)-HLC. After 6 h, NC-HLC still had not internalized a detectable level of DiI-LDL, as evidenced by the lack of fluorescence signal. After 24 h, a minimal DiI fluorescence signal was detected, suggesting internalization by a non-receptor-mediated mechanism or through the expected 5% LDLR activity of this mutation (Fig. 1A, top panel). In contrast, the C-HLC presented a far greater capacity for DiI-LDL internalization over this time frame (Fig. 1A, bottom panel). At 6 h, the C-HLC showed bright DiI fluorescence, which qualitatively increased over 24 h (Fig. 1A, bottom panel, middle and bottom rows). Representative confocal 3D image stacks of FH (3040) and LDLR-corrected (3040c) HLC show punctate cellular internalization, likely indicative of endosome-liposome localization (Fig. 1B). Together, this indicates that the corrected HLC are capable of internalizing LDL via receptor-mediated mechanisms, and that statin treatment produces a time-dependent LDL internalization increase.

To quantify restoration of depleted cholesterol, we repeated the previous assay and at 0 h treated the HLC with 10 mM methyl-β-cyclodextrin for 45 min to extract stored cholesterol (i.e. 0 h) (Mahammad and Parmryd, 2015). At 0 h, unlabeled LDL-C was added to the remaining cultures for 6 h and 24 h, after which samples were also collected. Cholesterol content was measured using Amplex Red quantification (Amundson and Zhou, 1999) and normalized to total protein (Fig. 1C). At 0 h after cyclodextrin treatment, the starting cellular cholesterol content for NC-HLC and C-HLC was 15.7 µM/(µg/µl) and 11.6 µM/(µg/µl), respectively, with no statistical difference, indicating that the starting cholesterol content was equivalent for both cell populations. Six hours after cyclodextrin treatment, the NC-HLC and C-HLC cholesterol content was 10.6 µM/(µg/µl) and 14.5 µM/(µg/µl), respectively, which was still not significantly different between the cell groups at this time point, nor different from the 0 h starting time point. It was only after 24 h that a statistically significant difference was quantified. At 24 h, the NC-HLC contained 21 µM/(µg/µl) cholesterol, which was not significantly different from that at 0 h and 6 h. However, C-HLC showed a statistically significant (*P*<0.05) increase in cellular cholesterol [to 61 µM/(µg/µl)] with treatment, compared to values at 0 h and 6 h as well as to NC-HLC. This demonstrates a quantitative increase in the ability of C-HLC to internalize cholesterol and that the receptor-corrected cells have the ability to overcome cyclodextrin-inhibited endocytosis (Imelli et al., 2004). Together, these data support the normalization of LDLR-mediated LDL-C endocytosis in FH differentiated HLC when the class II mutation is directly corrected.

### Distribution of LDLR in FH and LDLR-corrected HLC

The normal LDLR is synthesized in the ER as a partially glycosylated precursor of 120 kDa. Upon reaching the Golgi, N- and O-linked sugars are processed for a molecular mass of 160 kDa. This process is delayed or completely abolished when there are class II mutations, trapping the misfolded LDLR in the ER (Hobbs et al., 1990). Previous work studied fibroblasts containing the same homozygous 3-bp deletion in the LDLR as our patient FH cells using electron microscopy to identify the distribution of the intracellular LDLR (Pathak et al., 1988). Unlike normal fibroblasts, in which most LDLR was found in coated pits, the class II mutant cells had less than 5% of LDLR detectable on the cell surface, in coated pits or in vesicles in the endocytic pathway. Instead, most of the class II LDLR was present in membrane extensions of the rough ER (Pathak et al., 1988). We sought to examine whether NC-HLC retained the LDLR in the ER and whether correction resolved transport inhibition.

**Fig. 2.**
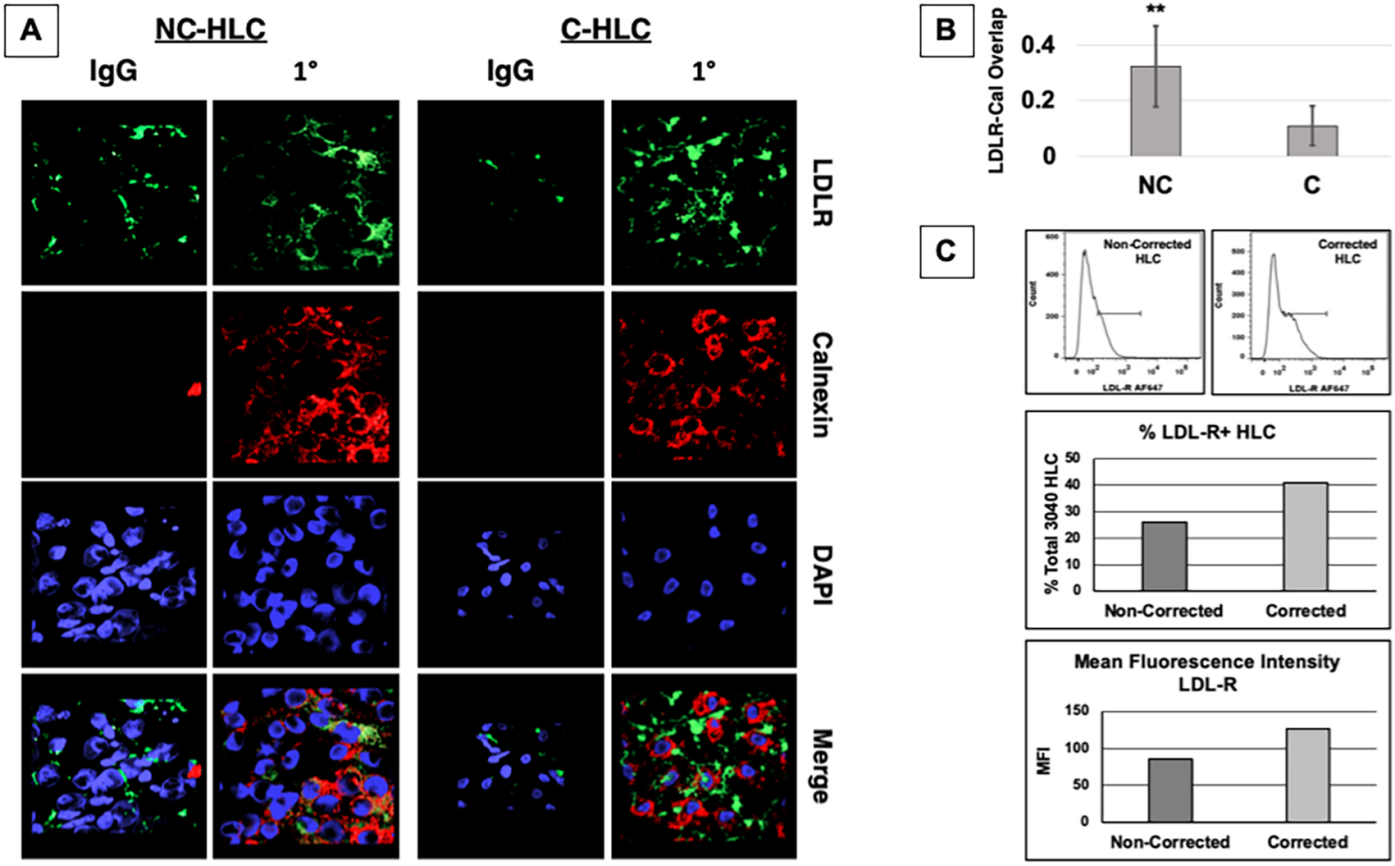
**LDLR colocalizes with** **calnexin-ER in FH HLC.** (A) After differentiation, HLC were treated overnight in LPDS medium supplemented with rosuvastatin followed by immunocytochemistry and confocal imaging, 100× oil immersion objective. AMIRA software was used to stack slices and merge channels to present an overview of localization of the LDLR (green), calnexin-ER (red) and nucleus (DAPI). (B) Quantification of colocalization using Fluoview software indicated that NC-HLC did have a significantly greater colocalization of the LDLR with calnexin than C-HLC. The graph values represent the mean±s.d. (*n*=5) per cell type from three experiments repeated in the laboratory. Statistics were performed using an unpaired two-tailed Student's *t*-test, **P<0.01. (C) Both non-corrected (NC) and corrected (C) cell lines were differentiated to HLC and assayed for surface LDLR expression using AF647-conjugated anti-LDLR antibody clone C7 (top). Cells were quantified for percentage total positive labeling (middle) and mean fluorescence intensity (MFI) (bottom).

As our ER marker, we selected the chaperone calnexin, which interacts with newly synthesized proteins, acting to retain misfolded proteins in the ER (Leach and Williams, 2003). After differentiation, HLC were treated overnight with LPDS and rosuvastatin, after which we examined protein localization using immunocytochemistry for calnexin and LDLR. We imaged the cells by confocal microscopy and 3D rendered the image stacks (Fig. 2A). NC-HLC showed a similar perinuclear labeling pattern between LDLR (green) and calnexin (red), suggesting colocalization. In the CRISPR-corrected HLC, confocal image stacks showed the same perinuclear labeling as in NC-HLC, but the LDLR labeling did not match that of calnexin, suggesting LDLR transport out of the ER. Using Fluoview Program software, images were further analyzed, utilizing the colocalization processing tool to quantify overlap Quantification of overlap confirmed that NC-HLC have significantly greater LDLR colocalization to the ER-calnexin (0.38) than C-HLC (0.09), and the difference was statistically significant by unpaired Student's *t*-test (**P<0.01) (Fig. 2B).

As already described, class II mutations cause misfolding, but some portion of the mutant protein can reach the cell surface, depending on the specific mutation (Li et al., 2004). We sought to quantify the amount of surface LDLR between 3040 (5% activity) and CRISPR-corrected, 3040c HLC using the C7 clone antibody, which recognizes an extracellular epitope in the LDL-binding domain (Beisiegel et al., 1982, 1981) (Fig. 2C). Basing our gate on the unlabeled control (Fig. S1), we found that the isotype control labeled 18.9% and 23.9% positive for non-corrected and corrected cells, respectively (Fig. S1). Cytometry using the C7 anti-LDLR antibody indicated that 26% and 41.1% of NC-HLC versus C-HLC were labeled, respectively (Fig. 2C, middle; Fig. S1). From the histograms, the mean fluorescence intensity (MFI) for the unlabeled control was 39.6, whereas that for IgG_2b_ NC-HLC and C-HLC was 70.7 and 87.8, respectively (Fig. S1). The C7 anti-LDLR antibody labeling for NC-HLC and C-HLC was 85.7 and 127, respectively (Fig. 2C, bottom). Together, these data confirm the ER localization of the misfolded LDLR and confirm that, with CRISPR correction, transport from the ER is restored.

### Rosuvastatin regulation of *LDLR* transcript and protein levels in FH and corrected cells

**Fig. 3.**
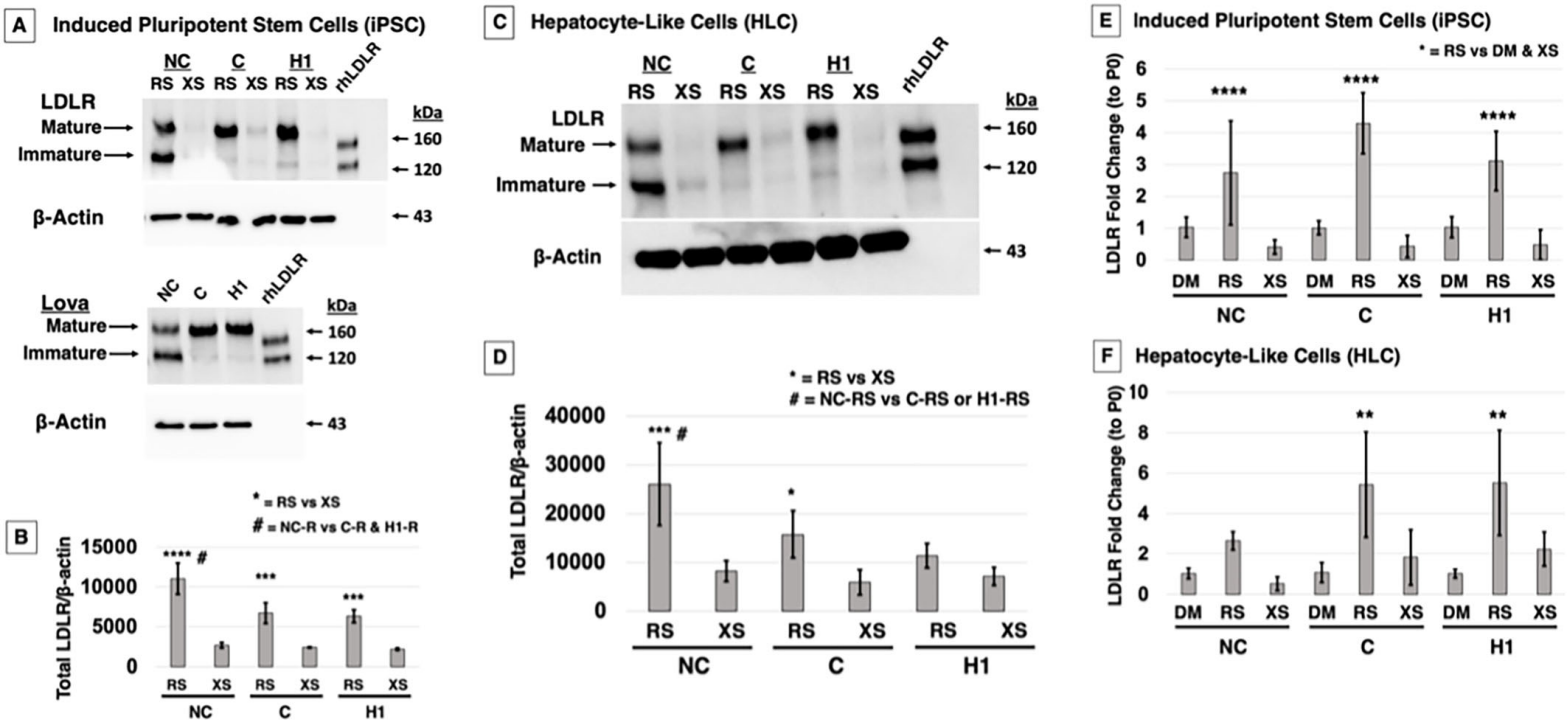
**Rosuvastatin increases total LDLR protein levels and an accumulation of immature LDLR in NC cells.** (A) PSC were treated overnight in LPDS medium supplemented with either lovastatin (Lova), rosuvastatin (RS) or excess sterols (XS). Western blot analysis for LDLR shows that NC-iPSC upregulate immature LDLR in Lova, whereas C-iPSC and H1-ESC express mature LDLR. RS treatment produces the same response, and LDLR is suppressed when exposed to sterols. Recombinant human LDLR protein (rhLDLR) was used as a detection control. (B) Quantification of total LDLR demonstrated that RS-treated NC-iPSC had a significantly greater total LDLR than C-iPSC and H1-ESC treated with RS. All three statin-treated cell types were significantly greater compared to their XS-treated counterparts. (C) iPSC/ESC were differentiated to HLC. Under the same conditions of rosuvastatin RS or XS treatment, NC-HLC express greater total LDLR, predominantly as immature protein, which is converted to all mature protein in C-HLC and H1-HLC. LDLR levels decreased with sterol treatment. (D) Two-way ANOVA analysis of total LDLR in HLC indicated a significant difference in mean between cell types and in response to RS treatment. Total LDLR in RS-treated NC cells was statistically different from that in RS-treated C and H1 cells. As expected, for each cell type, total LDLR was significantly different when comparing RS and XS treatments. (E) qPCR quantification of *LDLR* mRNA shows that RS treatment significantly increases *LDLR* transcript levels in NC-iPSC, C-iPSC and H1-ESC compared to DMSO (DM) control and XS treatment. (F) Similarly, HLC showed significant difference in mean *LDLR* mRNA with treatment and within cell type; in both C and H1 cells, means were different in response to RS treatment in comparison to DM and XS treatment, although no difference was detected within the NC cell treatment groups. The graph values represent the mean±s.d. (*n*=3) per treatment, per cell type of three experiments repeated in the laboratory using a two-way ANOVA Holm–Sidak post-hoc test. **P<0.01, ***P<0.001, ****P<0.0001, *^#^P*<0.05 between RS-treated cell types.

In our previously published work, we used lovastatin (5 µM) to induce LDLR expression and found that NC-iPSC almost exclusively expressed immature LDLR compared to C-iPSC, which converted protein to all mature LDLR (Omer et al., 2017). We also found that total LDLR (i.e. immature+mature) was greater in the NC-iPSC compared to C-iPSC. The LDLR receptor class II mutants are reported to be degraded via the proteasome pathway (Li et al., 2004), and lovastatin has been cited to inhibit or modulate the 20S proteasome pathway (Murray et al., 2002; Rao et al., 1999; Wójcik et al., 2000), although at doses of one to two orders of magnitude higher than those we used. We compared our results obtained with lovastatin to those obtained under the same conditions using rosuvastatin and excess sterols, which act independently of the LDLR and enter the cell via pinocytosis to decrease LDLR expression (Fig. 3A). A similar expression pattern was observed in response to lovastatin and rosuvastatin treatment, indicating that increased LDLR in NC-iPSC was not caused by lovastatin secondary inhibition of the proteasome pathway. Quantification of total LDLR indicated a significantly greater total LDLR in NC-iPSC than in C-iPSC and H1-ESC with rosuvastatin treatment, while C-iPSC LDLR was equivalent to that of normal control H1-ESC (Fig. 3B). Two-way ANOVA with a Holm–Sidak post-hoc test determined the significance of differences in total LDLR in all three PSC lines treated with rosuvastatin compared to excess sterols (Fig. 3B, ***P<0.001, ****P<0.0001), and in NC-iPSC treated with rosuvastatin compared to C-iPSC and H1-ESC treated with rosuvastatin (Fig. 3B, *^#^P*<0.001).

We differentiated the PSC to HLC, and, after treatment with rosuvastatin, we observed an induction of LDLR expression primarily in immature form in NC-HLC and very little mature LDLR (Fig. 3C). C-HLC and H1-HLC expressed solely mature LDLR (Fig. 3C). Excess sterols downregulated LDLR protein synthesis in all the cells as expected. Quantification and two-way ANOVA with a Holm–Sidak post-hoc test confirmed a rosuvastatin-induced upregulation of total LDLR protein mainly in the immature form in NC-HLC that was significantly greater than in the same cells in response to excess sterols. Rosuvastatin also induced more total LDLR protein in NC-HLC compared to treated C-HLC and H1-HLC (Fig. 3D, *P<0.01, ***P<0.001, ^#^*P*<0.05).

To investigate whether total LDLR protein and increase in immature LDLR (Fig. 3B,D) was due to differential transcriptional regulation of the LDLR between NC and C cells in the presence of statins, quantitative polymerase chain reaction (qPCR) analysis was used on both iPSC and HLC post-treatment with control carrier dimethyl sulfoxide (DMSO), rosuvastatin and excess sterols. For both iPSC and differentiated HLC, rosuvastatin increased *LDLR* transcript levels across all the cell types compared to excess sterols or DMSO control, and two-way ANOVA with a Holm–Sidak post-hoc test showed the differences to be significant (Fig. 3E,F, **P<0.01, ****P<0.0001). However, there was no difference in transcript levels when comparing statin treatment across cell lines or differentiation state, which is to be expected with statin treatment.

Taken together, these results show that both iPSC and HLC can be induced to express LDLR with statin treatment, and although the NC cells generally have more total LDLR that accumulates predominantly in the ER, this is not due to any difference in transcription. This suggests that the accumulating misfolded LDLR may be caused by an issue with protein ER processing or degradation.

### Treatment of cells with rosuvastatin does not induce ER stress

**Fig. 4.**
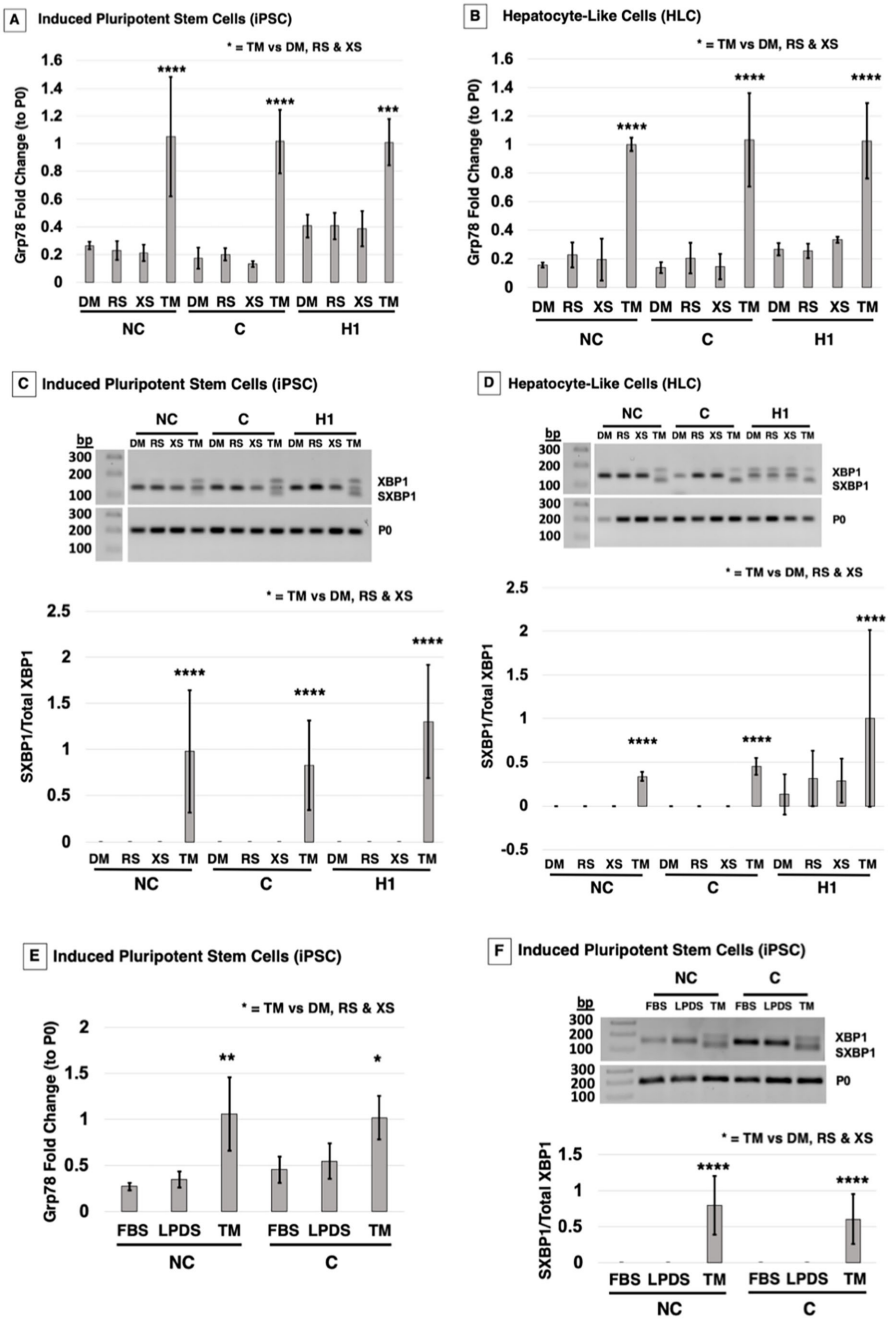
**FH NC cells do not activate the UPR** **following rosuvastatin treatment.** Following treatment with either carrier DM, RS, XS or tunicamycin (TM; 5 μg/ml for 4.5 h), cells were tested for ER stress and UPR markers at the mRNA level in iPSC/ESC and HLC. (A) qPCR analysis in iPSC indicated that *Grp78* mRNA levels do not change with RS treatment compared to DM/XS controls across the three cell groups. TM significantly increased *Grp78* mRNA in NC, C and H1 stem cells. (B) HLC presented the same trend, with TM significantly upregulating *Grp78* transcripts, whereas RS did not. (C) PCR for spliced *XBP1* (*SXBP1*) visualized on a 2% agarose gel indicated that TM treatment induced splicing of *XBP1* that was not present in RS-treated iPSC/ESC. Quantification of *SXBP1/XBP1* shows significant *SXBP1/XBP1* activation in TM-treated cells across all three cell types. (D) HLC also showed significant changes with treatment, and response to TM was significantly different from that to DM, RS and XS within cell lines, but no XBP1 cleavage was detected across the cell lines without TM treatment. (E) iPSC were treated with LPDS only, FBS only or LPDS with TM. qPCR quantification in iPSC indicated a *Grp78* mRNA level pattern that is significantly different in response to TM treatment, but not in response to LPDS or FBS treatment, across the three cell groups. (F) PCR for *SXBP1* indicated that TM treatment induced splicing of *XBP1* that was not present in LPDS- or FBS-treated iPSC/ESC. Quantification of *SXBP1/XBP1* shows significant *SXBP1/XBP1* activation in TM-treated cells across the three cell types. The graph values represent the mean±s.d. (*n*=3) per treatment, per cell type of three experiments repeated in the laboratory using a two-way ANOVA Holm–Sidak post-hoc test. *P<0.05, **P<0.01, ****P<0.0001 for TM treatment compared to all other treatments in each respective cell group.

Two studies using overexpression vectors in model cells reported that class II LDLR mutants are retained in the ER, causing ER stress and activating the UPR (Jørgensen et al., 2000; Sørensen et al., 2006). Because statins cause an accumulation of immature protein in NC-iPSC and HLC, we investigated statin treatment-induced ER stress in class II iPSC/HLC and whether it was normalized in the corrected cells. The ER chaperone Grp78 is a major factor involved in maintaining ER homeostasis. It is also the first component activated in the UPR. Little is known about the ER stress responses in iPSC or derived HLC; therefore, we used the ER stress inducer tunicamycin (TM) as a positive control. TM functions by inhibiting glycoprotein synthesis, inducing protein unfolding and activating the UPR. PSC/HLC were treated with either rosuvastatin, excess sterols or control DMSO overnight or TM for 4.5 h. We specifically looked at *Grp78* mRNA transcript levels as it has been documented that *Grp78* mRNA levels increase quickly in response to ER stress. In addition, in the reported overexpression class II LDLR mutant models, *Grp78* mRNA and protein levels are upregulated (Jørgensen et al., 2000; Sørensen et al., 2006). TM treatment significantly increased *Grp78* transcript levels across all cell types in both PSC and HLC, demonstrating that UPR pathways were capable of being activated (Fig. 4A,B). As expected, neither excess sterols, which downregulate *LDLR* transcription, nor DMSO had any effect on *Grp78* transcription. Surprisingly, after treatment with rosuvastatin, no quantifiable increase in *Grp78* expression in either PSC or HLC could be detected (Fig. 4A,B). Two-way ANOVA with a post-hoc Holm–Sidak test confirmed a significant upregulation of *Grp78* mRNA with TM treatment, but not with any other treatment (Fig. 4A,B, ***P<0.001, ****P<0.0001).

X-box binding protein 1 (XBP1) is a transcription factor that becomes activated in response to accumulation of unfolded proteins (Schröder and Kaufman, 2005). The splicing of a 26-nucletoide intron from *XBPI* generates the transcription factor spliced XBP1 [XBP1 (S)] (Hetz et al., 2011; Lee et al., 2002; Yoshida et al., 2001). XBP1 (S) regulates UPR genes for folding, ERAD, autophagy and organelle biogenesis (Hetz et al., 2011; Kakiuchi et al., 2006; Lee et al., 2002; Yoshida et al., 2001). The XBP1 (S) isoform can be detected through PCR, and, after TM treatment, PSC/HLC exhibited the XBP1 (S) isoform (Fig. 4C,D). However, no XBP1 splicing was detected with any other treatment in the NC-iPSC/NC-HLC or C-iPSC/C-HLC. Although H1-ESC did not show XPB1 splicing other than TM, curiously H1-HLC had a basal level of XBP1 (S), which was statistically equivalent to that with TM treatment. Quantification of XBP1 (S) to total XBP1 confirmed that rosuvastatin treatment did not activate XBP1 (S) or the UPR in NC, C or H1 cells (Fig. 4C,D). Two-way ANOVA with a Holm–Sidak post-hoc test showed that only TM treatment significantly activated the splicing of XBP1 in all iPSC/ESC) (Fig. 4C, ****P<0.0001). Although no significance was detected for induction of XBP1 splicing in HLC by ANOVA (*P*=0.09), this is likely due to variance in HLC differentiation (Fig. 4D).

The effects of statins on activating or inhibiting ER stress are unclear (Chen et al., 2008; Li et al., 2017). LPDS has long been shown to increase LDLR activity independent of the addition of statins (Goldstein and Brown, 1974). To determine if statins may be inhibiting ER stress and UPR activation, we asked whether LPDS medium induced ER stress, using TM and fetal bovine serum (FBS) as positive and negative controls, respectively. TM in LPDS medium induced *Grp78* mRNA transcription, as seen previously. However, there was no difference in *Grp78* transcripts between FBS- and LPDS-treated NC-iPSC or C-iPSC (Fig. 4E). The XBP1 (S) isoform was also only detected in the TM-treated iPSC; LPDS and FBS treatment did not activate splicing of XBP1 (Fig. 4F). Quantification and a two-way ANOVA with a Holm­­–Sidak post-hoc test of XBP1 (S) to XBP1 confirmed that TM treatment significantly activated the splicing of XBP1 in iPSC, but no significant treatment effect was seen with LPDS medium (Fig. 4F, ****P<0.0001). Altogether, these data show that although FH NC and C cells as well as normal H1 controls can be induced to activate ER stress and UPR with TM treatment, exposure to statins that cause downstream LDLR upregulation does not appear to cause ER stress and induce UPR, as has been reported by others.

## DISCUSSION

**Fig. 5.**
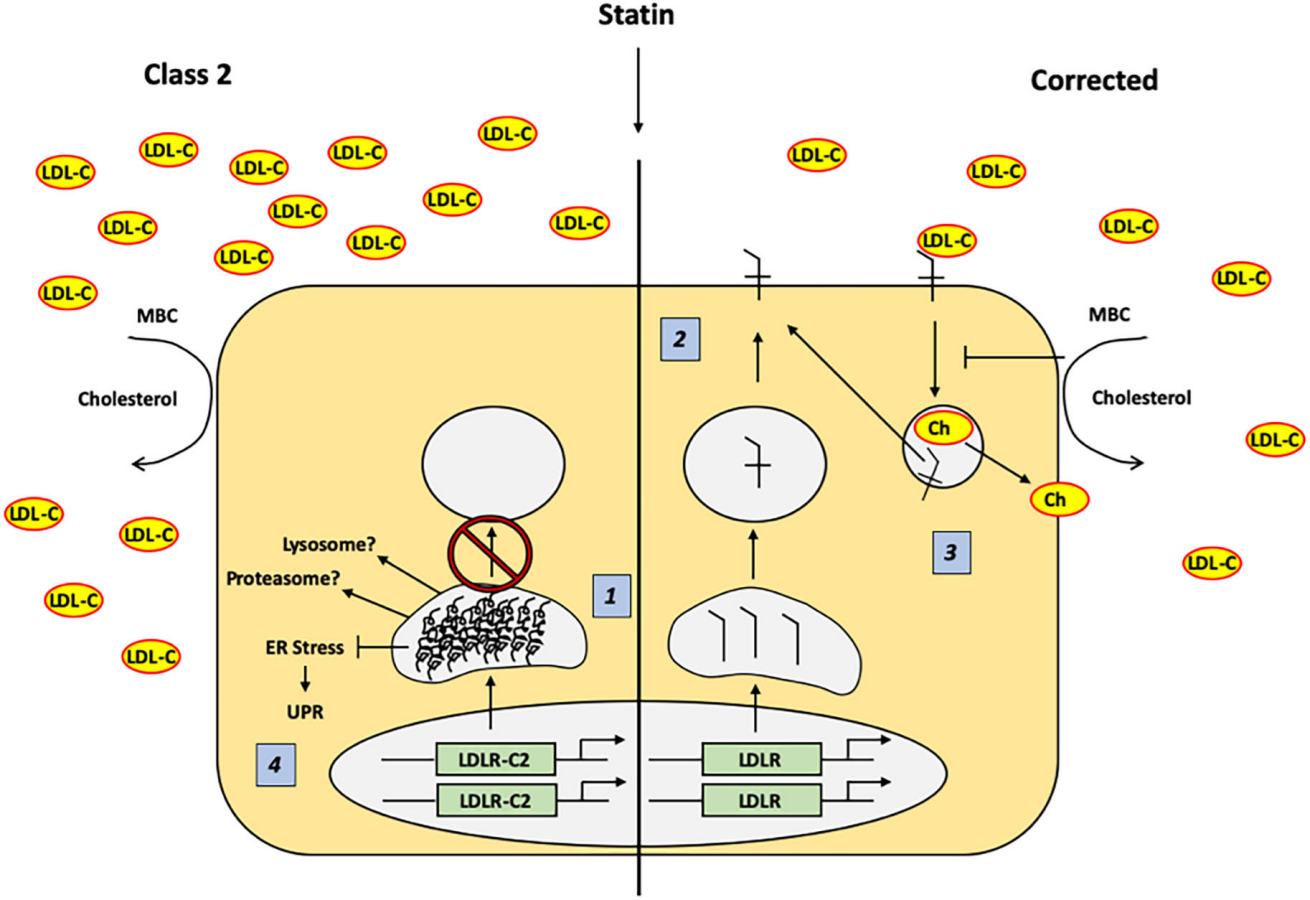
**Response to statin treatment in class II LDLR and corrected iPSC and HLC.** (1) Treatment with 5 μM RS causes uniform upregulation of class II and corrected *LDLR* gene transcription, but results in an accumulation of misfolded immature (120 kDa) LDLR protein as well as greater total LDLR (120 kDa+160 kDa) in class II compared to corrected cells. (2) Corrected LDLR is transported from the ER to the Golgi and on to the plasma membrane, where it participates in receptor-medicated LDL-C internalization. (3) When cellular cholesterol is removed by MBC treatment, LDLR-mediated endocytosis and recycling is inhibited, delaying LDL-C internalization kinetics. (4) Class II LDLR mutations accumulate as misfolded protein in the ER when treated with 5 μM rosuvastatin, but this accumulation does not appear to cause ER stress and induction of the UPR. Ch, cholesterol; ER, endoplasmic reticulum; LDL-C, low-density lipoprotein (LDL) cholesterol; LDLR, LDL receptor; LDLR-CII, class II LDL receptor; MBC, methyl-β-cyclodextrin; UPR, unfolded protein response.

The novel and important findings of this study are that (1) the mechanisms regulating LDL-receptor mediated endocytosis are restored in genetically corrected FH iPSC and HLC, and (2) statin-mediated accumulation of misfolded LDLR did not induce ER stress in class II FH iPSC or HLC with ∼5% LDLR activity. These data provide proof of concept that the FH mutant and genetically corrected iPSC and derived HLC could be useful as models to compare mutant to corrected cellular response, or perform drug testing or even cell-based therapy work (Cayo et al., 2012). Overexpression studies have reported that class II LDLR mutants activate the ER stress UPR pathway. In contrast to these results, induced overexpression of LDLR by statin treatment did not appear to cause ER stress or induce UPR (Fig. 5).

It has been hypothesized that class II mutations change the spacing between the highly conserved cysteine residues, thereby interfering with disulfide bond formation and proper LDLR folding (Russell et al., 1989). Retention of the class II mutant in the ER as an immature protein suggests that it is a transport problem (Yamamoto et al., 1986) or a protein folding disease (Gent and Braakman, 2004). We demonstrated previously that modification of the class II-causing genomic mutation permanently corrects the defect and allows for proper processing to the mature LDLR and receptor-medicated endocytosis (Omer et al., 2017). We also showed that statin treatment differentially caused accumulation of more total LDLR in the FH class II mutant (predominantly immature) compared to CRISPR-corrected (predominantly mature) cells (Omer et al., 2017). This led us to question why the mutant FH cells are retaining more total protein with statin treatment.

Most of our previous work was performed on cells in the pluripotent state; therefore, we initially investigated if receptor-medicated endocytosis was also normalized in differentiated HLC. A time course of internalization of DiI-labeled LDL-C showed that, even after 24 h, very little DiI could be detected in the mutant HLC, whereas robust fluorescence is detected in the corrected HLC at both 6 h and 24 h. When cellular cholesterol is depleted by statin and methyl-β-cyclodextrin, even at 6 h, neither FH nor corrected HLC showed a quantifiable change in cholesterol restoration. After 24 h, no statistical change in cellular cholesterol was quantified in NC-HLC. However, in corrected HLC, between 6 h and 24 h, LDLR activity appears to have been sufficient to restore cellular cholesterol levels, likely including within the plasma membrane (Imelli et al., 2004; Park et al., 1998).

Because the class II LDLR mutant is misfolded and retained in the ER, we next asked if we could detect differential LDLR localization between FH and corrected cells. In the NC-HLC, we observed LDLR colocalized with calnexin-ER that was absent in the C-HLC, supporting restored transport of the corrected LDLR (Fig. 2). Previous work with class II FH563 fibroblasts from the Dallas Collection and having the equivalent mutation to GM03040 were also carefully studied through electron microscopy to identify the distribution of the intracellular LDLR (Pathak et al., 1988). Less than 5% of LDLR was detectable on the cell surface and in coated pits or vesicles in the endocytic pathway. Instead, most of the class II LDLR was present in membrane extensions of the rough ER (Pathak et al., 1988). It is hypothesized that the extensions at which the mutant LDLR is localized is the site at which the ER is blocking exit of the LDLR, which appeared to be morphologically similar to the transitional zone of the ER implicated in transport of secretory proteins to the Golgi (Pathak et al., 1988). This correlates with our observation in the internalization assays (Fig. 1), in which minimal DiI fluorescence was visible in the NC-HLC.

One possibility for the accumulation of misfolded LDLR with statin treatment was the use of lovastatin (Omer et al., 2017). Lovastatin was the first US Food and Drug Administration (FDA)-approved statin drug and has since been found at high doses to inhibit the proteasome, which is reported to be the mechanism of FH class II LDLR degradation (Murray et al., 2002; Rao et al., 1999; Yamamoto et al., 1980). However, this effect is only seen in the closed-ring β-lactone form, which does not inhibit 3-hydroxy-3-methyl-glutaryl-coenzyme A (HMG-CoA) reductase (Rao et al., 1999). When we compared the effect on mutant LDLR accumulation of lovastatin and rosuvastatin, we detected the same increase in total protein levels of LDLR in NC cells, as well as similar total mature protein between corrected LDLR and wild-type control, which demonstrates that lovastatin proteasome inhibition was not causing the misfolded protein accumulation (Fig. 3). The presence of immature LDLR in FH cells has been reported previously (Yamamoto et al., 1980). A pulse-chase study comparing unaffected and class II FH fibroblasts (the same mutation found in our FH iPSC) showed that the LDLR of normal fibroblasts had been transported to the Golgi for processing to the mature 160-kDa proteins, while 95% of the class II LDLR remained in the 120-kDa form (Yamamoto et al., 1980). The accumulation of mutant LDLR compared to the corrected LDLR was not the result of differential transcriptional control, which is what we expected because the cells were treated with statins to inhibit HMG-CoA reductase and upregulate LDLR (Endo et al., 1976, 1977). We also found that a similar accumulation of mutant LDLR occurs in NC-iPSC and differentiated NC-HLC, indicating that mutant LDLR accumulation is a post-translational regulation mechanism. FH class II LDLR proteins are reported to be degraded by two mechanisms: proteasome (Li et al., 2004; Melman et al., 2002) and lysosome (Zelcer et al., 2009) degradation. It is possible that one or both of these systems is overwhelmed by the statin-mediated expression induction, allowing misfolded protein to accumulate, but then one would expect this to cause ER stress and UPR induction (Gardner et al., 2013; Harding et al., 2003).

It has been demonstrated that ER chaperones retain misfolded class II LDLR in the ER. Two class II mutant LDLRs, C646Y and W556S, were retained in the ER and bound to Grp78 when overexpressed in model Chang cells, as shown through mass spectrometry and western blotting (Jørgensen et al., 2000). This was confirmed in Chinese hamster ovary (CHO) cells overexpressing two other class II LDLR mutations, in which there was prolonged binding of Grp78 to mutant LDLR (Sørensen et al., 2006). In addition, ER stress activation in CHO cells overexpressing mutant G544V LDLR was observed. Spliced *XBP1* mRNA was present in the class II LDLR mutant and absent in overexpressed wild-type LDLR. Class II mutant LDLR overexpression resulted in a fold induction of phosphorylated PERK (Sørensen et al., 2006). In contrast, we did not observe an increase in *Grp78* expression (Fig. 4A,B) or XBP1 splicing (Fig. 4C,D) in NC cells treated with rosuvastatin. Our study used iPSC and derived HLC rather than an overexpression model. It is possible that statins are causing a low-grade chronic ER stress that we were unable to detect. A study of liver samples from untreated patients with chronic hepatitis identified ‘ER-stressed hepatocytes’ in clusters scattered in the liver parenchyma presented with protein expression of ATF-6, IRE1α and PERK. However, qPCR of UPR genes did not show induction of ER stress; instead, genes involved in inflammation and apoptosis were significantly upregulated in these patient samples (Asselah et al., 2010). Another case study dissected whether liver hepatotoxicity was due to statins or another cause (Russo et al., 2014). Prolonged latency was seen in patients taking atorvastatin, simvastatin, fluvastatin and rosuvastatin, and they presented both hepatocellular and cholestatic patterns of liver injury. Under these conditions, statin-induced liver injury took months to years to become clinically relevant (Russo et al., 2014).

Two other possibilities must be considered for explaining the differential results between previous reports on class II mutants and our findings: expression induction and statin inhibition of ER stress-induced UPR. The principal report indicating that class II misfolded proteins cause ER stress utilized CHO cells stably transfected with tetracycline-inducible overexpression vectors (Sørensen et al., 2006). It is likely that, even with statin treatment, our cells did not produce the same levels of LDLR as found in the overexpressing CHO cells, which could account for the differential ER stress response. This begs the question, can expression at physiological levels of class II mutant LDLR cause ER stress, even with statin treatment, or is it the overexpression that causes ER stress? In our report, all cells depended on two alleles for LDLR production, while the pcDNA4-based plasmids used by Sørensen and colleagues are driven by a cytomegalovirus (CMV) promoter, and, although the number of copies per cell are not specified, it is probably more than two. The other possibility is statin inhibition of ER stress-induced UPR (Mollazadeh et al., 2018). Statins are reported to have both inhibitory (Li et al., 2017; Xu et al., 2016) and stimulatory (Morck et al., 2009; Wang et al., 2017) effects on ER stress and UPR. It is possible that, in our case, statin treatment is playing an inhibitory role. The FH iPSC and HLC are capable of UPR induction by TM, indicating a functional UPR system. However, the TM-treated cells were also cultured in the presence of statins, but were capable of UPR stimulation. Additionally, the iPSC cultured in LPDS and TM without statin had a similar quantitative effect on UPR marker induction compared to statin-pretreated cells. If statins are inhibiting class II UPR induction, it could be clinically relevant. This could suggest that class II FH patients may suffer from misfolded LDLR accumulation and ER stress, but statin therapy blocks downstream induction of UPR. To conclude, these data validate that an FH corrected cell line can physiologically mediate LDLR endocytosis and present a suitable model to study class II LDLR. In addition, we have determined that the UPR is not activated in a physiological model of class II LDLR iPSC and HLC.

## MATERIALS AND METHODS

### Ethics

All experimental elements and usage of recombinant DNA were approved by the University of Louisville's Institutional Biosafety Committee prior to initiation of the project (IBC 14-043).

### Cell culture and hepatocyte differentiation

Human iPSC (derived from Coriell Cell Repository GM03040 fibroblasts), were cultured on hESC-qualified Matrigel-coated plates (BD Biosciences, San Jose, CA, USA) in mTeSR1 (STEMCELL Technologies, Vancouver, Canada) with the medium changed daily. H1 cells (WA01/NIH 0043, WiCell, Madison, WI, USA) were cultured as the iPSC. iPSC and H1 cells are collectively referred to as PSC. GM03040 iPSC/HLC that retain the FH class II mutation are referred to here as non-corrected (NC), whereas CRISPR-corrected GM03040 iPSC/HLC are referred to as corrected (C). All derived cells have been authenticated and tested for contamination (Omer et al., 2017).

Briefly, PSC were plated on hESC-qualified Matrigel-coated 60-mm plates at 5×10^5^ cells. The next day, stage 1 definitive endoderm differentiation was initiated by replacing mTeSR1 with stage 1 differentiation medium [RPMI1640 (Thermo Fisher Scientific), B27 (1×, Thermo Fisher Scientific)] supplemented with human activin A (100 ng/ml, Peprotech, Rocky Hill, NJ, USA) and human WNT3A (50 ng/ml; R&D Systems, Minneapolis, MN, USA). Medium was changed daily for 5 days. This was followed by a 5-day culture in stage 2 hepatoblast medium [knockout Dulbecco's modified Eagle's medium (KO-DMEM), 20% knockout serum replacement (KSR), 0.5× GlutaMAX, 1% non-essential amino acids, 0.1 mM β-mercaptoethanol and 1% DMSO (v/v) (all Thermo Fisher Scientific)] with medium replacement every other day. Cells were finally cultured in stage 3 hepatocyte maturation medium for 11 days [HepatoZYME (Thermo Fisher Scientific) with 10 µM hydrocortisone 21-hemisuccinate (Sigma-Aldrich), 1× GlutaMAX supplemented with human hepatocyte growth factor (HGF; 10 ng/ml, Peprotech) and human oncostatin M (OSM; 20 ng/ml, Peprotech)]. The medium was changed every other day.

### qPCR analysis

PSC or HLC were cultured overnight in 5% LPDS (Thermo Fisher Scientific) medium alone or supplemented with either 5 µM rosuvastatin (EMD Millipore, Burlington, MA, USA) or excess sterols (10 µg/ml cholesterol and 5 µg/ml 25-hydroxycholesterol; Sigma-Aldrich). Positive control cells were treated with 5 µg/ml TM (Thermo Fisher Scientific) for 4 h or FBS (Thermo Fisher Scientific) overnight. Control cells were treated with DMSO overnight. At the end of treatment, PSC and HLC were lysed using 150 µl of 0.1% β-mercaptoethanol in RLT Buffer (Qiagen, Valencia, CA, USA). The lysates were purified with Qiashredder and RNeasy kits (Qiagen) according to the manufacturer's instructions. RNA was quantified with a NanoDrop One Spectrophotometer (Thermo Fisher Scientific). cDNA was synthesized using 1 µg RNA with SuperScript IV reverse transcriptase (Thermo Fisher Scientific) in a 20 µl volume. qPCR was performed using Fast SYBR Green Master Mix (Thermo Fisher Scientific) with primers obtained from Integrated DNA Technologies (Table S1). Reactions were run on the StepOnePlus Real-Time PCR System (Thermo Fisher Scientific). Raw data were quantified in Microsoft Excel and statistics performed in GraphPad Prism 8 (La Jolla, CA, USA). PCR was carried out for spliced *XBP1* and *XBPI* expression with PCR Supermix (Thermo Fisher Scientific). Amplicons were evaluated via 2% agarose gels (Bio-Rad, Hercules, CA, USA). Then, 10 µl of amplicons were added to 2 µl 6× loading buffer (Thermo Fisher Scientific). Gels were run at 80 V for 60 min and imaged via a ChemiDoc Imaging System with Image Lab Touch Software (Bio-Rad).

### Western blotting

PSC or HLC were treated as described above. Following treatment, the cells were thoroughly washed with PBS^+/+^ prior to adding 200 µl RIPA lysis buffer (Thermo Fisher Scientific) plus protease inhibitor cocktail (Thermo Fisher Scientific) for cell collection. Lysates were rocked overnight at 4°C followed by centrifugation for 15 min. Supernatants were used for protein quantification by DC Protein Assay (Bio-Rad). Then, 0 µg/sample of total protein was run on 4-10% mini-protean TGX precast gels (Bio-Rad) at 200 V for 40 min. Proteins were transferred onto PVDF (Bio-Rad) then blocked in 3% milk/PBST. Membranes were probed overnight with the anti-LDLR antibody [1:1000 in 5% bovine serum albumin (BSA)/PBST; R&D Systems] or β-actin (1:1000 in 5% BSA/PBST; Santa Cruz Biotechnology, Dallas, TX) overnight at 4°C. The membranes were incubated in either horseradish peroxidase (HRP)-conjugated bovine anti-goat IgG H+L (Jackson ImmunoResearch, West Grove, PA, USA) or anti-mouse IgG (Cell Signaling, Danvers, MA, USA), HRP-linked antibodies (1:5000 in 3% milk/PBST) the following day for 1 h at room temperature. Clarity Max Western ECL Blotting Substrate (Bio-Rad) was used to visualize the proteins on the Bio-Rad Imager. Densitometry was performed using Bio-Rad imaging software. A list of all antibodies used for analyses is presented in Table S2.

### Cellular cholesterol replenishment

Cells were plated on 35-mm tissue culture dishes and differentiated until day 1 of stage three, as described above. Cells were treated with LPDS medium supplemented with 5 µM rosuvastatin for 48 h. After 48 h, cells were treated with LPDS medium supplemented with 5 µM rosuvastatin and 10 mM methyl-β-cyclodextrin (Sigma-Aldrich) for 45 min. After 45 min (time point 0 h), cells were collected as described below. The remaining dishes were cultured with LPDS medium supplemented with 5 µM rosuvastatin and 10 µg/ml LDL-C (Thermo Fisher Scientific). Cells were collected at 6 h and 24 h.

Cells were collected by incubating in TrypLE Express (Thermo Fisher Scientific) for 5 min then gently scraping and transferring cells to a 15-ml tube. After centrifugation (200 ***g***, 4 min), the cell pellet was resuspended in 200 µl chloroform/methanol (2:1 v/v) mixture, vortexed and centrifuged (14,000 ***g***, 5 min) to allow separation into three layers. A micropipette was used to carefully discard the top aqueous layer containing RNA. Next, a micropipette was used to gently push past the interphase layer (a thin membrane of protein) to reach the organic-phase layer containing the lipids. This bottom lipid layer was transferred into a new microcentrifuge tube. The lipid solution was dried using the Savant SpeedVac Plus vacuum (Thermo Fisher Scientific) for 30 min. The dried lipids were resuspended in 1× reaction buffer supplied in the Amplex Red Cholesterol Assay Kit (Thermo Fisher Scientific). The middle protein layer was resuspended in RIPA lysis buffer, incubated overnight at 4°C and processed the next day for protein analysis.

Collected lipid content was analyzed with the Amplex Red Cholesterol Assay Kit as per the instructions. Readings were measured on the Synergy4 spectrophotometer (BioTek, Winooski, VT, USA) with Gen5 software (BioTek) at an excitation of 560 nm and emission detection at 590 nm. Lipid content was normalized to total protein level as measured by DC protein assay.

### Fluorescence-labeled LDL uptake assay

NC-iPSC and C-iPSC were plated in three of four wells of a four-well chamber slide and differentiated to HLC (see above). Three wells were treated overnight in LPDS medium supplemented with 5 µM rosuvastatin. The following day, two wells in rosuvastatin were treated with 10 µg/ml DiI-LDL (Thermo Fisher Scientific) for 6 h or 24 h while the remaining well did not receive any DiI-LDL. Cells were fixed with 2% paraformaldehyde (PFA)/PBS (10 min, 24°C; Electron Microscopy Sciences, Hatfield, PA, USA) and mounted with VECTASHIELD Antifade Mounting Medium with DAPI (Vector Laboratories, Burlingame, CA, USA). Slides were imaged using an Olympus IX81 fluorescence or FV1000 confocal microscope (Center Valley, PA, USA).

### Flow cytometry

For flow cytometry, 1.5-4×10^5^ corrected or non-corrected 3040 iPSC-derived hepatocytes were resuspended in 100 μl flow wash buffer [1% BSA (Sigma-Aldrich) in Dulbecco's PBS (Thermo Fisher Scientific), 0.1% sodium azide (Sigma-Aldrich), 10 mM Hepes (Sigma-Aldrich) and 2 mM EDTA (Thermo Fisher Scientific)]. Then, 0.1 μl Fc block (BD Biosciences, Franklin Lake, NJ, USA) was added to each suspension and incubated in the dark at room temperature for 10 min. Cells were then labeled with Alexa Fluor 647-conjugated anti-LDLR antibody (C7 clone; Novus Biologicals) or mouse IgG_2b_ isotype control (Novus Biologicals) according to the manufacturer's instructions (1 h at 4°C, protected from light) or left unlabeled. Following incubation, cells were washed twice with flow wash buffer. LDLR expression data were collected on a BD LSRII cytometer (BD Biosciences) and analyzed with FlowJo software (BD Biosciences).

### Immunocytochemistry and image analysis

Following rosuvastatin treatment overnight in LPDS medium, HLC were fixed with 2% PFA/PBS (10 min, 24°C), permeabilized with 0.05% Triton X-100/PBS (10 min, 24°C; Sigma-Aldrich) and washed with PBS. Cells were then blocked with 5% normal donkey serum/PBS (Jackson ImmunoResearch) for 1 h followed by blocking with an avidin/biotin blocking kit (Vector Laboratories). Primary antibodies were diluted in 5% donkey serum/PBS and incubated on cells overnight at 4°C. Secondary antibodies diluted in 5% donkey serum/PBS (1:1000) were added to the cells (2 h, 24°C) followed by washing and mounting with VECTASHIELD Antifade Mounting Medium with 4′,6-diamidino-2-phenylindole (DAPI). Slides were imaged using an Olympus BX61WI confocal microscope with Fluoview (FV10-ASW 4.1, Olympus). The three channels were merged into a single image using AMIRA software (Thermo Fisher Scientific) (Ramakrishnan et al., 2016). Using Fluoview (FV10-ASW 4.1), confocal image stacks were analyzed utilizing the colocalization processing tool to both visualize overlapping of images as well as quantification of overlap. A list of all antibodies used for analyses is provided in Table S2.

### Statistical analysis

Data from a minimum of three independent experiments were analyzed via two-tailed Student's *t*-test or by two-way ANOVA with a post hoc using Holm–Sidak multiple comparison test in SigmaPlot v14 and graphs generated in Excel. Data are presented as mean±s.d. Figure legends contain further details.

## Supplementary Material

Supplementary information
