# Supplementary material for: Familial hypercholesterolemia class II low-density lipoprotein receptor response to statin treatment
Source: Dis Model Mech. 2020 Apr 6;13(4):dmm042911. doi: 10.1242/dmm.042911 (PMC7157586; doi:10.1242/dmm.042911)
Supplement: Supplementary information [file dmm-13-042911-s1.pdf]

Supplementary Fig. 1

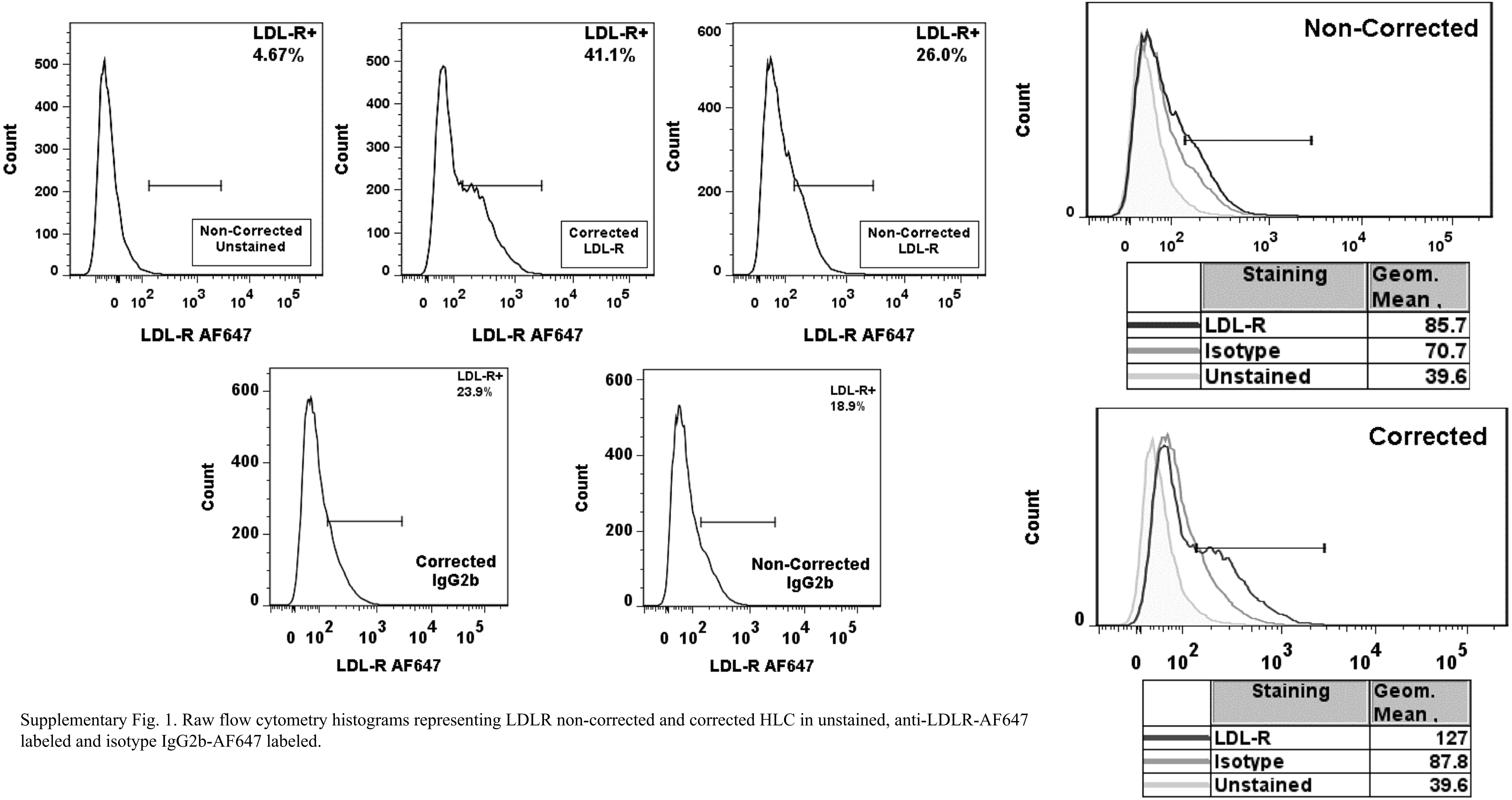

Supplementary Fig. 1. Raw flow cytometry histograms representing LDLR non-corrected and corrected HLC in unstained, anti-LDLR-AF647 labeled and isotype IgG2b-AF647 labeled.

Table S1 List of Primers Used in qPCR/PCR

Gene	Primer 1 (5' → 3')	Primer 2 (5' → 3')	Product Size (bp)
**LDLR**	GCAGTGTGACCGGGAATATGA	GTTGGTCCCGCACTCTTTGA	115
**GRP78**	CCGTTCAAGGTGGTTGAAAAGAA	TGGCGTTGGGCATCATTAAAA	200
**(S) XBP1**	CCTGGTTGCTGAAGAGGAGG	GGCAGGCTGCTGTCCTCAT	150; 124
**P0**	TCGACAATGGCAGCATCTAC	ATCCGTCTCCACAGACAAGG	200

Table S2 List of Antibodies and Fluorophores

Antigen (Catalog#)	Lot #	Dilution	Distributor
LDLR (AF2148)	NA	1:100 (ICC), 1:1000 (WB)	R&D Systems, Minneapolis, MN
Calnexin (MAB3126)	2919754	1:100	Millipore, Billerica, MA
Beta-Actin (sc-69879)	G1517	1:1000	Santa Cruz Biotechnology, Dallas, TX
gIgG (NBP1-97057)	NA	1:100	Novus Biologicals, Littleton, CO
mIgG (10400C)	SG255128	1:100	Invitrogen, Carlsbad, CA
Alexa Fluor Donkey Anti-Goat 488 (A11055)	1869589	1:1000	Invitrogen
Alexa Fluor Donkey Anti-Mouse 546 (A10036)	1832039	1:1000	Invitrogen
HRP-Bovine Anti-Goat IgG H+L (805-035-180)	138694	1:5000	Jackson ImmunoResearch, West Grove, PA
Anti-Mouse IgG, HRP-linked (7076S)	33	1:5000	Cell Signaling, Danvers, MA
LDLR-AF647 (C7) (NBP1-78159AF647)	A-3-042519-AF647	1:50	Novus Biologicals
IgG2b-AF647 (NBP2-27228)	AB100711A-7-042519-AF647	1:83	Novus Biologicals
